# Macrophage pyroptosis in atherosclerosis: therapeutic potential: Pyroptosis in atherosclerosis

**DOI:** 10.3724/abbs.2025004

**Published:** 2025-02-14

**Authors:** Jianying Ma, Yixian Wang, Wenna Xu, Hanjing Wang, Zhengdong Wan, Jiawei Guo

**Affiliations:** 1 Department of Vascular and Endovascular Surgery the First Affiliated Hospital of Yangtze University Jingzhou 434000 China; 2 Department of Pharmacology School of Medicine Yangtze University Jingzhou 434023 China; 3 Department of Interventional Jingzhou Hospital Affiliated to Yangtze University Jingzhou 434020 China

**Keywords:** atherosclerosis, macrophage, pyroptosis

## Abstract

Atherosclerosis (AS) is a chronic inflammatory disease characterized by the accumulation of lipid-rich plaques in arterial walls, leading to cardiovascular events such as myocardial infarction and stroke. Macrophage pyroptosis, a form of programmed cell death driven by the NLRP3 inflammasome and caspase-1 activation, plays a critical role in the progression and destabilization of atherosclerotic plaques. This review explores the molecular mechanisms underlying macrophage pyroptosis and their significant contributions to AS pathogenesis. Recent advancements have highlighted the therapeutic potential of targeting key components of the pyroptotic pathway, including the use of nanotechnology to increase drug delivery specificity. These strategies are promising for reducing inflammation, stabilizing plaques, and mitigating the clinical impact of AS. Future studies should focus on translating these findings into clinical applications to develop effective treatments that can halt or reverse AS progression by modulating macrophage pyroptosis.

Atherosclerosis (AS) is a chronic, progressive inflammatory disease of the medium and large arteries characterized by the accumulation of lipid-rich foam cells and fibrous tissues within the intima of the elastic arteries. This accumulation can lead to the development of plaques and serious complications, such as myocardial infarction and stroke [1]. Many risk factors, such as age, smoking, unhealthy diet, and dyslipidemia, have been identified as contributing factors to the incidence of AS [2]. AS progression is characterized by dysregulated programmed death of plaque cells, including vascular endothelial cells (VECs), macrophages, and vascular smooth muscle cells (VSMCs), which exacerbates inflammatory responses [ 3, 4] . In the early stages of AS, oxidized low-density lipoprotein (ox-LDL) induces injury, activation, and death in VECs, facilitating the recruitment of monocytes and other circulating leukocytes for transendothelial migration. Within the endothelium, monocytes differentiate into macrophages, which become foam cells upon engulfment of excess lipids [5]. Additionally, VEC death promotes the proliferation and migration of adjacent VSMCs into the arterial intima, enhancing the synthesis of extracellular matrix components and thereby contributing to plaque formation and impairing vasodilatory functions [6]. Although macrophage death may initially help suppress inflammation in early atherosclerotic lesions, it significantly accelerates disease progression in the advanced stages of AS [7]. The inadequate clearance of dying macrophages within vessel walls leads to the release of intracellular pro-inflammatory cytokines and lipids into the extracellular space, driving necrotic core formation and contributing to plaque instability [ 7, 8] . These studies suggest that macrophage death is a critical factor in AS progression. 

Macrophages undergo various types of cell death [8]. In advanced atherosclerotic plaques, large necrotic cores are resulted primarily from regulated forms of necrotic cell death. During necrosis, the intracellular contents of dying cells are released into the extracellular space, creating a highly inflammatory environment [9]. Necrosis can occur accidentally or be triggered through tightly regulated pathways such as necroptosis, pyroptosis, and ferroptosis [10]. Pyroptosis, a form of regulated necrosis initiated by inflammasomes and characterized by the formation of plasma membrane pores, plays a crucial role in plaque progression [11]. Pyroptosis exacerbates plaque instability by releasing pro-inflammatory cytokines and intracellular contents, further enlarging the necrotic core and serving as a pivotal driver in the progression of AS [12]. Even with optimal treatment using antihypertensive and cholesterol-lowering agents, including statins, a significant residual cardiovascular risk persists in AS patients [13]. This has driven the exploration of novel therapeutic strategies to address the underlying inflammatory mechanisms that contribute to plaque progression and instability. Given the critical role of macrophage pyroptosis in AS pathogenesis, the modulation of this form of cell death may emerge as a promising new target for therapeutic intervention. 

This review focuses on key advances in understanding the molecular mechanisms of pyroptosis, the relationship between macrophage pyroptosis and AS, and the potential of targeting macrophage pyroptosis as a therapeutic approach for treating AS.

## Mechanistic Insights into Pyroptosis Pathways

**Figure 1:**
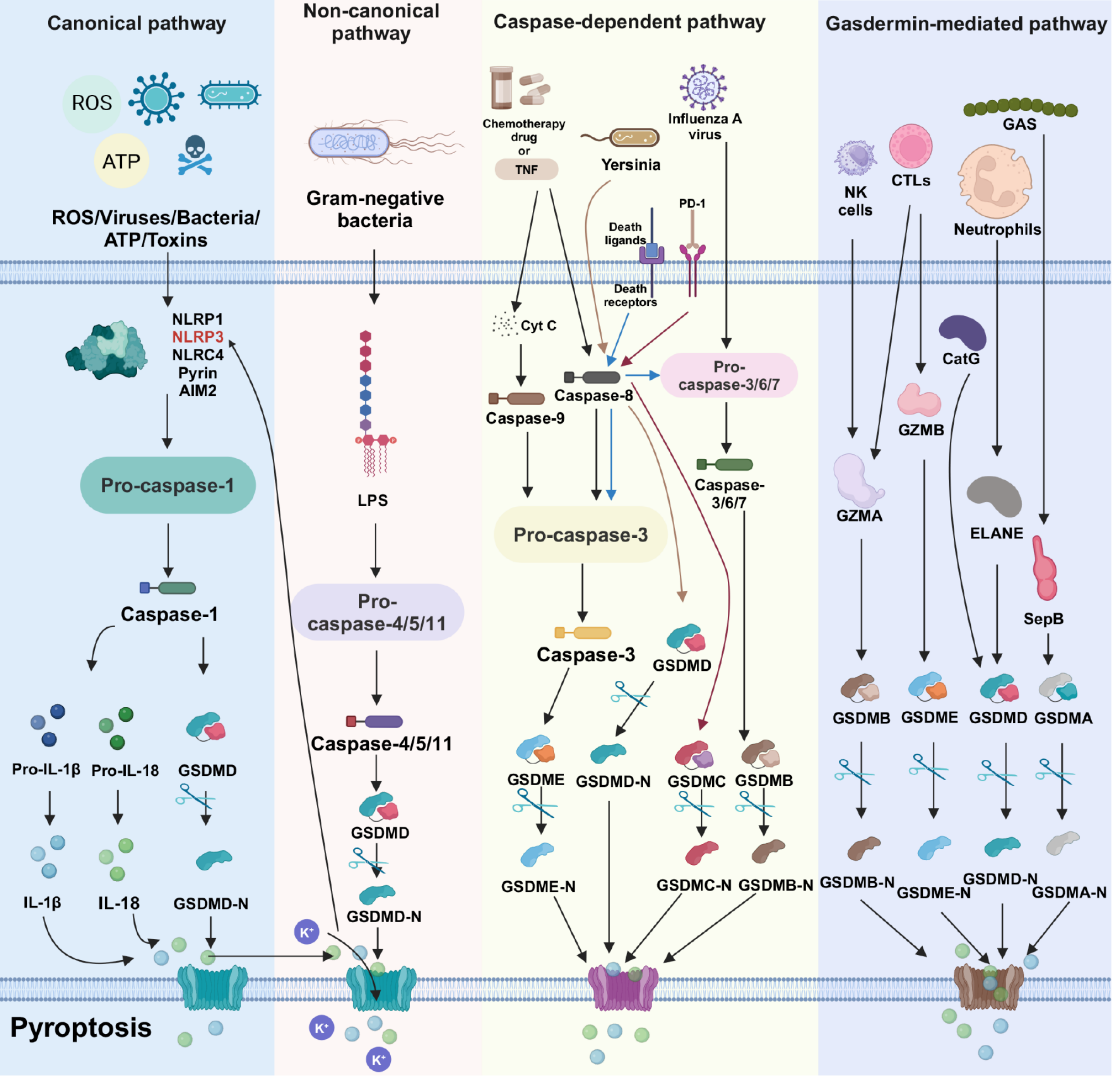
Figure 1 The basic mechanisms of pyroptosis This figure outlines the four key pathways driving pyroptosis. The canonical pathway is triggered by inflammasome activation, leading to the caspase-1-dependent cleavage of gasdermin D (GSDMD), which in turn facilitates the release of interleukin-1β (IL-1β) and interleukin-18 (IL-18). In the noncanonical pathway, lipopolysaccharide (LPS) directly activates caspases-4/5/11, inducing GSDMD cleavage and subsequent pyroptosis. The caspase-dependent pathways are characterized by caspase-3-mediated cleavage of gasdermin E (GSDME) and caspase-8-mediated cleavage of both gasdermin D (GSDMD) and gasdermin C (GSDMC). Furthermore, caspases-3/6/7 cleave gasdermin B (GSDMB), collectively contributing to pyroptotic cell death. Finally, the gasdermin-mediated pathways are modulated by a variety of proteases, including granzyme A (GZMA), granzyme B (GZMB), neutrophil elastase (ELANE), cathepsin G (CatG), and streptococcal pyrogenic exotoxin B (SpeB), which cleave distinct gasdermins, leading to membrane pore formation and the subsequent pyroptosis of target cells.

Pyroptosis has recently emerged as a distinct form of regulated cell death characterized by intricate and interwoven signaling pathways. We have detailed the canonical, non-canonical, caspase-dependent, and gasdermin-driven pathways ( Figure 1). Ongoing research may reveal additional mechanisms underlying pyroptosis.

### Canonical pathway: inflammasome and caspase-1 activation

The canonical pyroptotic pathway is driven primarily by the activation of caspase-1 via inflammasome assembly [14]. The inflammasome is a multiprotein complex comprising pattern recognition receptors (PRRs), an adaptor protein called apoptosis-associated speck-like protein (ASC), and pro-caspase-1 [15]. Five distinct PRRs (NLRP1, NLRP3, NLRC4, Pyrin, and AIM2) assemble into inflammasomes via the canonical pathway [14]. Notably, NLRP1 and NLRC4 distinguish themselves from other inflammasomes because of their unique ability to either engage with caspase-1 directly or utilize ASC as a mediator of caspase-1 activation, depending on the specific activation context [16]. Studies have demonstrated that both NLRP1 and NLRC4 possess caspase activation and recruitment domains (CARD), enabling them to directly engage with the CARD of pro-caspase-1, thereby facilitating activation in an ASC-independent manner [ 17, 18] . The NLRP3 inflammasome comprises NLRP3, ASC, and pro-caspase-1. Its activation occurs through a two-step process: the first step involves the transcriptional upregulation of NLRP3, pro-caspase-1, and IL-1β, while the second step involves the assembly of NLRP3 with ASC and pro-caspase-1 [19]. The assembly of the NLRP3 inflammasome is initiated in response to infectious and non-infectious inflammatory triggers, including bacterial and viral components, extracellular adenosine triphosphate (ATP), particulate matter, reactive oxygen species (ROS), crystalline structures, and membrane-disrupting toxins [ 20, 21] . Extracellular ATP, particulate substances, crystalline deposits, and membrane-disrupting toxins are widely recognized as key enhancers of NLRP3 inflammasome activation [22]. Pro-caspase-1 undergoes hydrolysis into the p10 and p20 subunits and is converted into its active form, caspase-1, following the activation of the PRR by various stimuli [23]. Caspase-1 activation leads to the cleavage of gasdermin D (GSDMD) into GSDMD-N and GSDMD-C fragments while concurrently converting pro-IL-1β and pro-IL-18 into their active cytokines, IL-1β and IL-18 [24]. GSDMD-N disrupts the integrity of the cell membrane by forming non-selective pores that permit an uncontrolled flux of ions and water, ultimately causing osmotic imbalance and cellular swelling. This event facilitates the release of active IL-1β and IL-18, ultimately resulting in pyroptosis [25]. 

### Non-canonical pathway: lipopolysaccharide recognition and caspase-4/5/11 in pyroptosis

Unlike the canonical pathway, the non-canonical pathway functions independently of inflammasomes. Human caspase-4/5 and mouse caspase-11 are activated through direct interactions between their N-terminal CARD and cytoplasmic lipopolysaccharide, a component derived from gram-negative bacteria, such as *Escherichia coli* [ 26, 27] . Once activated, caspase-4/5/11 cleaves GSDMD into GSDMD-N, which then oligomerizes and forms pores in the plasma membrane, initiating pyroptosis [28]. Caspase-4/5/11 does not directly cleave pro-IL-1β or pro-IL-18. However, the cleavage of GSDMD leads to potassium ion efflux, thereby activating the NLRP3/caspase-1 pathway, which ultimately results in the release of mature IL-1β and IL-18 through the pores, culminating in pyroptosis [ 24, 29] . Additionally, caspase-11-mediated pannexin-1 cleavage promotes ATP release, activates P2X7 receptors, and amplifies NLRP3 inflammasome signaling [30]. 

### Caspase-dependent pathways: the role of apoptotic caspases in pyroptosis

Initially, apoptosis-related caspases were not thought to induce pyroptosis. However, emerging evidence suggests that these caspases also play a role in pyroptosis at the molecular level. Although caspase-3 typically drives apoptosis, its activation can trigger pyroptosis under conditions of elevated gasdermin E (GSDME) expression. Wang *et al* . [31] reported that caspase-3, once activated by apoptotic stimuli, such as chemotherapeutic drugs, induces pyroptosis by cleaving GSDME, thereby releasing a pore-forming fragment that disrupts the cell membrane, leading to pyroptotic cell death [31]. Another study revealed that the formation of the Apaf-1 apoptosome by cytochrome c leads to the activation of caspase-9, which subsequently activates caspase-3. Additionally, caspase-8, which is activated through death receptor signaling, can also activate caspase-3. In both pathways, active caspase-3 cleaves GSDME at Asp270 to produce a GSDME-N fragment, which triggers pyroptosis by forming pores in the plasma membrane [32]. 

Additionally, Yersinia can suppress TGFβ-activated kinase 1 in mouse macrophages through its effector YopJ, subsequently utilizing the lysosomal Rag-Ragulator complex as a platform to recruit the adaptor Fas-associated death domain, receptor-interacting protein kinase 1 (RIPK1), and pro-caspase-8. This cascade leads to the phosphorylation of RIPK1 and the activation of caspase-8, which induces GSDMD cleavage and triggers pyroptosis [ 33– 35] . Programmed death ligand 1 interacts with the death receptor to shift TNFα-induced apoptosis towards pyroptosis in cancer cells, thereby promoting tumor necrosis. Following TNFα stimulation, caspase-8 selectively cleaves gasdermin C (GSDMC) to generate the N-terminal fragment GSDMC-N, which perforates the cell membrane, initiating pyroptosis [36]. Importantly, binding of the death ligand to its cognate death receptor triggers the formation of a death-inducing signaling complex, leading to the activation of caspase-8. This activation subsequently results in the cleavage and activation of the executioner caspases-3, -6, and -7 [37]. Despite its activation by the influenza A virus, the caspase-3/6/7 cascade is recognized as an apoptosis-associated pathway [38]. Chao *et al*. [39] proposed that gasdermin B (GSDMB) is cleaved by caspases-3, -6, and -7, leading to the release of an N-terminal GSDMB-N fragment. This fragment has the potential to form pores in the cell membrane, which can trigger pyroptosis. These findings indicate that apoptosis-related caspases play a crucial role in determining the balance between pyroptosis and apoptosis, highlighting the need for further investigation into the underlying mechanisms [39]. 

### Gasdermin-mediated membrane pore formation: triggers and consequences

The gasdermin family, comprising gasdermins A, B, C, D, E, and DFNB59, is characterized by its ability to form pores in the cell membrane, which play a crucial role in the process of pyroptosis [40]. Molecular dynamics simulations have provided insight into the structural dynamics of GSDMD pores. These studies revealed that GSDMD N-terminal fragments oligomerize at the plasma membrane, forming β-sheet structures that create water-filled pores approximately 10-20 nm in diameter. These pores enable the release of pro-inflammatory cytokines, such as IL-1β and IL-18, contributing to the inflammatory cascade associated with pyroptosis [41]. Recent studies have demonstrated that gasdermin is directly cleaved by streptococcal pyrogenic exotoxin B (SpeB) and various serine proteases, such as granzymes, neutrophil elastase (ELANE), and cathepsin G (CatG). This cleavage triggers pyroptosis, which challenges the traditional view that this form of cell death is solely caspase-dependent [ 42– 45] . ELANE and CATG are serine proteases. Zhou *et al*. [42] demonstrated that granzyme A (GZMA) secreted by cytotoxic lymphocytes cleaves GSDMB, leading to pyroptosis in target cells. These findings revealed that cytotoxic T lymphocytes (CTLs) and natural killer cells utilize perforin to transport GZMA into target cells, where GZMA cleaves GSDMB at the Lys244 interdomain linker, subsequently inducing pyroptosis [42]. This cleavage event leads to the rapid oligomerization of GSDMB N-terminal fragments, which integrate into the plasma membrane, where they form pores that disrupt cellular ion homeostasis, eventually leading to osmotic lysis and cell death [46]. Second, Zhang *et al*. [47] reported that granzyme B (GZMB), a serine protease secreted by CTLs, cleaves GSDME at a site targeted by caspase-3, thereby inducing pyroptosis. Third, ELANE, a neutrophil-specific serine protease released from primary granules, is crucial for mediating inflammatory responses [48]. Kambara *et al*. [44] reported that aging neutrophil granulocytes bypass the caspase-mediated pathway of GSDMD activation by utilizing ELANE to cleave GSDMD upstream of the caspase cleavage point. This cleavage bypasses the canonical caspase pathway, highlighting the critical alternative pathway for GSDMD activation. This non-canonical activation pathway ensures that neutrophils can undergo pyroptosis even without caspase activity, thus maintaining their inflammatory response capabilities during chronic infections or aging [49]. ELANE-mediated cleavage generates a fully active GSDMD-N fragment that is subsequently released from cytoplasmic granules into the cytosol, triggering pyroptotic cell death [44]. Burgener *et al*. [45] reported that CatG, a serine protease, directly cleaves GSDMD to yield the stable N-terminal fragment GSDMD-p30. This fragment forms functional membrane pores that disrupt cell integrity and promote rapid cell lysis independent of caspase-1 activation. These findings suggest that CatG-mediated GSDMD cleavage is an additional mechanism for pyroptosis, particularly in cells where canonical inflammasome pathways are inactive or impaired [50]. This study further revealed that this cleavage occurs at Leu-274, two residues upstream of the classical caspase cleavage site, highlighting the pivotal role of CatG in driving pyroptotic cell death through an alternative pathway [45]. These findings suggest that serine proteases, including GZMA, GZMB, ELANE, and CatG, act directly on gasdermins and cleave them to produce active fragments that initiate pyroptosis. 

Group A Streptococcus (GAS), an important pathogen in skin infections, secretes the cysteine protease SpeB. A study by Deng *et al*. [51] demonstrated that SpeB specifically targets and cleaves gasdermin A (GSDMA) at Gln246 within its linker region. This cleavage liberates the N-terminal fragment, GSDMA-N, which subsequently oligomerizes and integrates into the cellular membrane, forming pores that induce pyroptosis [51]. SpeB-mediated cleavage bypasses caspase-dependent pathways and directly activates GSDMA, leading to rapid pore formation. This highlights how bacterial pathogens exploit host cell death mechanisms to enhance infection and immune evasion [52]. LaRock *et al*. [53] demonstrated that the virulence factor SpeB, which is secreted by GAS, specifically cleaves GSDMA in keratinocytes. Cleavage occurs within the disordered loop of GSDMA, producing a potent N-terminal fragment (GSDMA-N) that is translocated to the plasma membrane. Once integrated, GSDMA-N forms lytic pores, leading to membrane permeabilization and inducing pyroptosis [53]. Similarly, Zhao *et al*. [43] demonstrated that the cysteine protease SpeB, which is secreted by *Streptococcus pyogenes*, induces pyroptosis in keratinocytes by cleaving GSDMA at Gln246. The resulting N-terminal fragment (GSDMA-N) relocates to the plasma membrane, where it assembles into lytic pores, leading to cell death [43]. These findings illustrate the sophisticated strategies employed by pathogens such as GAS to induce pyroptosis and promote their own survival by hijacking gasdermin-mediated cell death pathways [41]. In general, SpeB, which is secreted by GAS, directly cleaves GSDMA, bypasses caspase activation, and triggers pyroptosis. 

## The Role of Macrophage Pyroptosis in AS

The pathogenesis of AS is a multifaceted process involving several key events, such as endothelial cell (EC) dysfunction, foam cell formation, VSMC migration and proliferation, and the secretion of pro-inflammatory cytokines. Among these factors, the role of macrophage pyroptosis is particularly significant, as it not only exacerbates local inflammation but also contributes to the destabilization of atherosclerotic plaques. Pyroptosis, triggered by the activation of the NLRP3 inflammasome and subsequent caspase-1 activation, leads to the release of pro-inflammatory cytokines such as IL-1β and IL-18. This cascade intensifies the inflammatory milieu within the plaque, promoting the further recruitment of immune cells, enhancing lipid accumulation, and accelerating plaque vulnerability. The resulting plaque instability increases the risk of rupture and thrombosis, underscoring the critical role of macrophage pyroptosis in AS [54]. 

### Macrophage pyroptosis and AS

During the early stages of AS, macrophages act as scavengers, eliminating modified lipoproteins and apoptotic cells to maintain plaque stability. However, as plaques progress, these macrophages transition into a pro-inflammatory state and ultimately undergo pyroptosis, a highly inflammatory form of cell death that significantly contributes to plaque destabilization and the risk of rupture in advanced atherosclerotic lesions [ 8, 55] . Studies have demonstrated that the primary expression of NLRP3 inflammasome components is localized within macrophages [56]. Consequently, elucidating the precise mechanisms underlying macrophage pyroptosis in AS may reveal novel therapeutic avenues and offer significant potential for the development of targeted interventions for the management of this disease. 

In 2010, a pioneering study provided the first evidence that cholesterol crystals trigger NLRP3 inflammasome activation in macrophages. This process is mediated by the induction of lysosomal membrane rupture, which facilitates the release of cathepsin proteins into the cytosol, ultimately leading to inflammasome assembly and activation [57]. Silencing of *NLRP3* eliminates the secretion of inflammatory cytokines triggered by cholesterol crystals, highlighting the pivotal role of the NLRP3 inflammasome in mediating this immune response [58]. These findings suggest that macrophage pyroptosis plays a critical role in the progression of AS. 

Ox-LDL is recognized as another key pathogenic contributor to AS. Research has demonstrated that ox-LDL significantly enhances the production of ROS within macrophages, a process that not only leads to cellular lysis but also triggers the release of inflammatory cytokines through the activation of the NLRP3 inflammasome [59]. Another study demonstrated that ox-LDL primes the NLRP3 inflammasome by engaging Toll-like receptors (TLRs), such as TLR2 and TLR4 [60]. These findings indicate that ox-LDL alone can induce macrophage pyroptosis by functioning as both a priming and an activation signal for the NLRP3 inflammasome. Unlike LDL, high-density lipoprotein (HDL), widely recognized for its anti-atherogenic and anti-inflammatory properties, exerts a protective effect by attenuating cholesterol crystal-induced activation of the NLRP3 inflammasome, thereby mitigating subsequent inflammatory responses, including pyroptosis. This protective action involves the downregulation of key inflammasome components, such as pro-IL-1β and NLRP3, and a reduction in caspase-1 activation, which are critical for the initiation of pyroptosis. By inhibiting these pathways, HDL effectively reduces inflammation and cell death via pyroptosis [61]. 

Activation of NLRP3 inflammasomes in macrophages triggers pyroptosis, which results in the release of pro-inflammatory cytokines such as IL-1β. This enhances lipid accumulation in macrophages, ultimately driving their transformation into foam cells, a key step in AS [62]. Inhibition of the NLRP3 inflammasome reduces foam cell formation in THP-1 macrophages by decreasing ox-LDL uptake and promoting cholesterol efflux [63]. Ox-LDL-induced IL-1β activates sterol regulatory element-binding protein-1, a transcription factor that regulates lipid synthesis and homeostasis, leading to increased lipid accumulation and foam cell formation. This process is mediated by the upregulation of key enzymes, including 3-hydroxy-3-methylglutaryl-CoA reductase (HMGCR) and fatty acid synthase (FAS) [64]. Hypertriglyceridemia is a recognized risk factor for AS. Triglycerides induce macrophage pyroptosis by activating caspase-1, thereby contributing to atherosclerotic plaque formation [65] .

Lysophosphatidylcholine (LPC), a key pro-inflammatory lipid derived from phospholipid hydrolysis, plays a significant role in mediating inflammatory processes and is crucial in the pathogenesis of AS [62]. Previous studies have demonstrated that LPC is a priming and activating signal for the NLRP3 inflammasome, inducing macrophage pyroptosis through mechanisms involving lysosomal damage and potassium efflux [62]. Oxidized phosphatidylcholines (oxPAPC), generated by phospholipid oxidation, are potent pro-inflammatory lipids that contribute to AS by triggering inflammatory pathways, including those leading to macrophage pyroptosis and activating key receptors on macrophages [66]. 1-palmitoyl-2-(5-oxovaleroyl)-sn-glycerophosphocholine, an endogenously generated oxPAPC, triggers IL-1β release in macrophages by increasing mitochondrial ROS production, leading to NLRP3 inflammasome activation [66]. 

In addition to damage‐associated molecular patterns, certain less-known pathogenic factors drive the progression of AS by inducing macrophage pyroptosis. Extensive epidemiological data suggest an association between periodontal disease and cardiovascular disease, although the precise mechanisms remain incompletely understood [ 67, 68] . TLRs play complex roles in *Porphyromonas gingivalis* (Pg), a key pathogen in human periodontal disease [ 69, 70] . TLRs are involved in pyroptosis and AS, suggesting a potential link between Pg infection and AS. In mouse models, Pg infection accelerates AS without significantly altering serum lipid metabolism. Further studies revealed that Pg exposure induces macrophage pyroptosis via a CD36-TLR2-dependent pathway [71]. Fine particulate matter (PM2.5) also plays an important role in AS [72]. Under ambient PM2.5 exposure, ApoE ^−/−^ mice presented an increased atherosclerotic lesion burden, marked by elevated levels of CD36 and NLRP3 inflammasome components in both the circulation and the aorta. Additionally, NLRP3 and caspase-1 activities are significantly increased in splenic macrophages, suggesting that macrophage pyroptosis contributes to the worsening of AS following exposure to PM2.5 [73]. 

Other studies suggest that, in addition to NLRP3, other inflammasomes, such as NLRP1, NLRC4, and AIM2, are crucial in AS. AIM2 regulates plaque vulnerability by promoting the release of IL-1β and IL-18, particularly in advanced disease stages. NLRP1 and NLRC4 are upregulated in coronary stenosis and contribute to systemic inflammation, whereas NLRP1, rather than NLRP3, drives the pro-inflammatory state in ECs, highlighting its role in AS [ 74– 76] . Advanced atherosclerotic plaques are marked by numerous dead cells, and nuclear dsDNA is a crucial product released from these cells. This dsDNA activates the AIM2 inflammasome in macrophages, leading to the release of pro-inflammatory cytokines, which in turn contribute to the destabilization of atherosclerotic lesions [74]. 

Ninjurin-1 (Ninj1) is a novel substrate of MMP9 that produces a soluble form with anti-inflammatory effects that can reduce AS [77]. Recent research has demonstrated a marked increase in Ninj1 expression in the aortic macrophages of ApoE ^−/−^ mice. Additionally, elevated levels of the soluble form of Ninj1 (sNinj1) have been detected in the serum of patients with AS; sNinj1 acts as an anti-inflammatory mediator and attenuates monocyte recruitment and atherogenesis. The role of Ninj1 in modulating macrophage pyroptosis within atherosclerotic lesions suggests a potential mechanism by which sNinj1 influences plaque stability and disease progression [77]. 

Noncoding RNAs (ncRNAs) are pivotal regulators of gene expression. ncRNAs are categorized according to their length into small ncRNAs such as microRNAs, small interfering RNAs, and long ncRNAs (lncRNAs) [78]. Sinapic acid (SA), a natural antioxidant, suppresses macrophage pyroptosis by downregulating the expression of the lncRNA MALAT1. Inhibition of MALAT1 reduces the activation of the NLRP3 inflammasome, thereby mitigating pyroptotic cell death in macrophages. These findings suggest a potential mechanism by which SA protects against AS progression by inhibiting macrophage pyroptosis [79]. Circular RNAs, characterized by their covalently closed-loop structures, constitute an emerging class of ncRNAs; however, their roles in AS remain unclear. Recent studies have indicated that interferon regulatory factor-1 (IRF-1) enhances macrophage pyroptosis in AS by downregulating circ_0029589 via increased m ^6^A modification. This modulation of circ_0029589 by IRF-1 may contribute to the progression of AS by promoting inflammatory cell death in macrophages [80]. 

In conclusion, macrophage pyroptosis is a pivotal process in AS progression, bridging the inflammatory response with plaque destabilization. The intricate interplay between NLRP3 inflammasome activation and various pathogenic stimuli, including ox-LDL and cholesterol crystals, underscores the critical role of macrophages in both the early and advanced stages of this disease. Understanding these molecular mechanisms not only deepens our understanding of AS but also highlights potential therapeutic targets that could mitigate plaque instability and reduce AS risk.

### Interactions among macrophages, endothelial cells, and vascular smooth muscle cells in pyroptosis and their role in AS

The progression of AS is characterized by intricate and sustained interactions among macrophages, ECs, and VSMCs, driven by a complex network of inflammatory mediators [81]. Although the precise mechanisms of these interactions have not yet been fully elucidated, it has been proposed that these cells communicate through microparticles (MPs), which are small vesicles released during pyroptosis [82]. MPs, which originate from the cell membrane, have been shown to facilitate the transport of paracrine and endocrine signals, thereby enabling intercellular communication under precise regulatory control [83]. This intercellular exchange mediated by MPs not only amplifies inflammatory cascades but also contributes to the destabilization of atherosclerotic plaques. By promoting crosstalk among macrophages, ECs, and VSMCs, MPs play a pivotal role in linking pyroptosis to the progression and exacerbation of AS, ultimately influencing disease outcomes [82]. MPs serve as vehicles for active substance transport. During macrophage pyroptosis, GSDMD and active caspase-1 are encapsulated within MPs. This packaging facilitates the transfer of pyroptotic signals to other cells, such as ECs, thereby promoting intercellular communication [84]. The interaction between pyroptotic macrophages and other cell types via these MPs may play a crucial role in the progression of AS by amplifying inflammatory responses and contributing to plaque instability [84]. These MPs induce pyroptosis by transferring active caspase-1 from macrophages to neighboring cells, including VSMCs and ECs. In these recipient cells, even at low GSDMD levels, caspase-1 can induce pyroptosis, thus contributing to the spread of inflammation and cellular damage within atherosclerotic lesions [85]. Furthermore, oxLDL-activated monocytes, upon differentiation into macrophages, promote EC pyroptosis through the activation of caspase-1 and the secretion of IL-1β. This inflammatory response exacerbates endothelial dysfunction and contributes to the progression of vascular injury [86]. Monocytes, which can differentiate into macrophages, play a crucial role in vascular inflammation by activating the NLRP3 inflammasome in VSMCs. This activation contributes to the characteristics of the inflammatory cascade in AS. The interaction between macrophages and VSMCs through inflammasome signaling highlights the intricate cellular dynamics that exacerbate vascular dysfunction and promote disease progression [87]. Recent findings indicate that IL-1β, produced during the interaction between macrophages and VSMCs, plays a crucial role in modulating VSMC functions related to adhesion, inflammation, and apoptosis. These functional alterations can be reversed through activation of the STAT3 signaling pathway. Inflammatory macrophages contribute to the early stages of AS by promoting the proliferation, migration, and transdifferentiation of VSMCs, thereby exacerbating disease progression [88] .

Overall, the critical role of pyroptosis in mediating the interactions among macrophages, ECs, and VSMCs in AS is evident. Through the release of MPs and the activation of key inflammatory pathways, such as the NLRP3 inflammasome and IL-1β signaling pathways, these cells engage in a complex network of cross-talk that drives disease progression. The involvement of pyroptosis in these cellular interactions underscores its importance in exacerbating vascular inflammation and plaque instability and, ultimately, in the advancement of atherosclerotic lesions.

## Therapeutic Potential of Targeting Macrophage Pyroptosis

**Figure 2:**
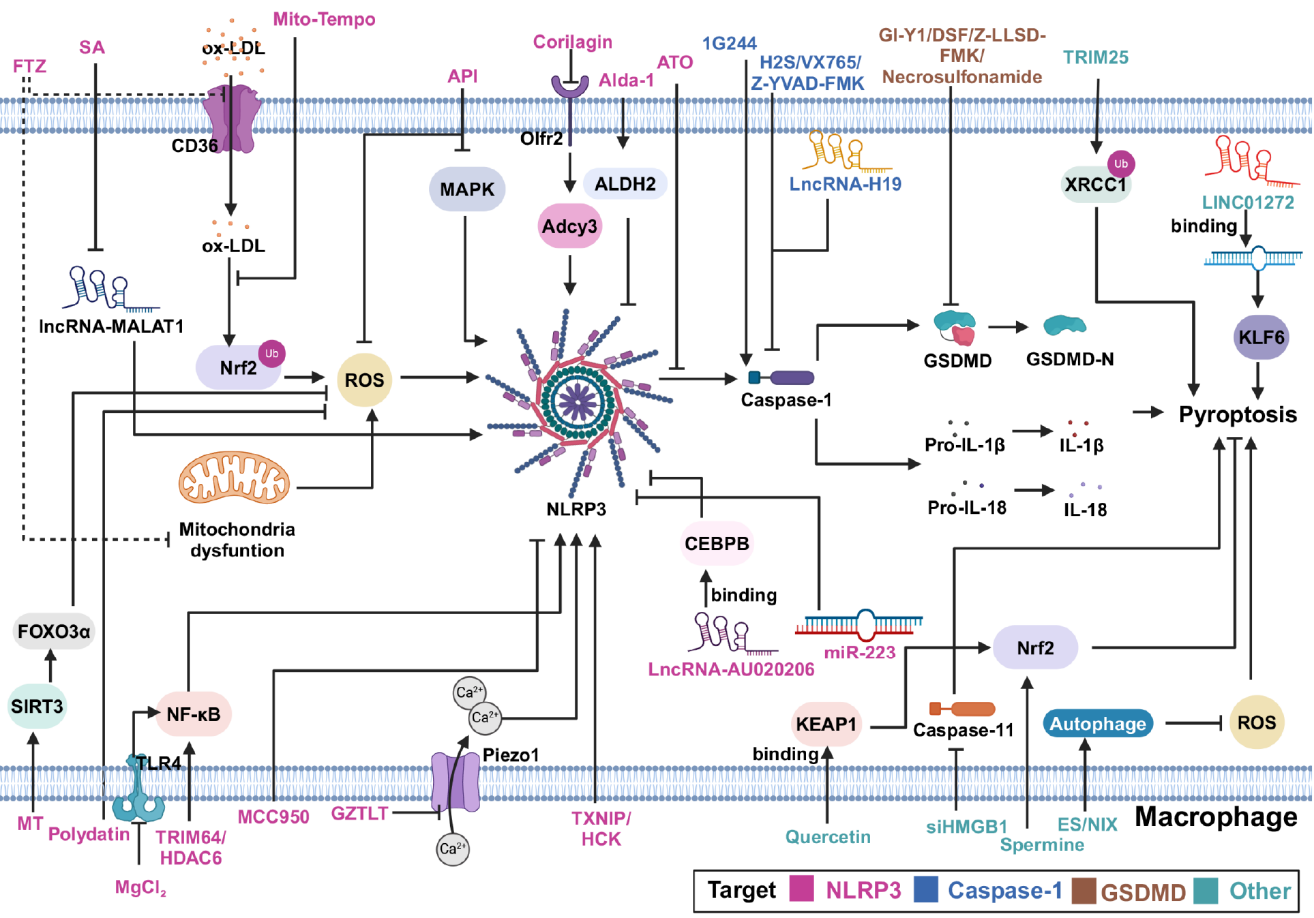
Figure 2 Schematic representation of therapeutic strategies targeting macrophage pyroptosis in atherosclerosis ATO, arsenic trioxide. GZTLT, Guizhitongluo Tablet. FTZ, Fufang Zhenzhu Tiaozhi. MT, melatonin. API, apigenin. HCK, hematopoietic cell kinase. SA, Sinapic acid. H2S, hydrogen sulfide. DSF, disulfiram. NSA, necrosulfonamide. ES, electrical stimulation.

In summary, we comprehensively elucidated the molecular mechanisms underlying macrophage pyroptosis and its critical role in AS. This insight is invaluable for investigating the pathogenesis of AS and identifying the key components of pyroptosis, such as the NLRP3 inflammasome, caspase-1, and GSDMD. Additionally, this knowledge will aid in advancing our understanding of therapeutic approaches targeting AS ( Figure 2 and Table1).

### NLRP3-related therapeutic targets

The NLRP3 inflammasome, a pivotal cytosolic PRR, is activated by a diverse array of stimuli [23]. Under normal conditions, the NLRP3 inflammasome maintains metabolic homeostasis. This dysfunction can trigger autoimmune and metabolic diseases. In AS, abnormal NLRP3 activation accelerates plaque formation and vascular damage by enhancing inflammation and cholesterol crystal accumulation [ 120, 121] . Mito-Tempo, a mitochondrion-targeted antioxidant, inhibits macrophage pyroptosis in AS by activating Nrf2 and suppressing NLRP3 inflammasome activation. This reduces caspase-1 and GSDMD cleavage, limiting the release of proinflammatory cytokines and thereby decreasing foam cell formation and slowing atherosclerotic plaque progression [89]. Despite these promising effects, clinical translation faces challenges such as optimizing dosing to minimize off-target effects and addressing patient variability in mitochondrial ROS levels. Further investigations in diverse models are essential for clinical advancement. Arsenic trioxide (ATO) downregulates the NLRP3 inflammasome by inhibiting CD36-mediated ox-LDL uptake and TLR4/NF-κB signaling, thereby reducing caspase-1 activation and IL-1β release, which ultimately suppresses macrophage pyroptosis and inflammation, slowing the progression of atherosclerotic plaques [90]. Preclinical trials have shown promise in reducing atherosclerotic plaque progression without significant toxicity at therapeutic doses. However, challenges such as optimizing its systemic bioavailability and mitigating potential toxicity in long-term use remain significant barriers to its clinical application. Corilagin inhibits NLRP3 inflammasome activation via the Olfr2 pathway in macrophages, thereby reducing caspase-1 cleavage and GSDMD-mediated pyroptosis. This decreases IL-1β and IL-18 release, thereby limiting inflammation and slowing AS progression [91]. Guizhitongluo tablet (GZTLT) inhibits macrophage pyroptosis by modulating the Piezo1/NLRP3 pathway, reducing calcium influx, caspase-1 activation, and IL-1β release [92]. Fufang Zhenzhu Tiaozhi (FTZ) inhibits macrophage pyroptosis by downregulating the activation of the NLRP3 inflammasome, which reduces caspase-1 activity and IL-1β release while also mitigating oxidative stress and preventing mitochondrial dysfunction in macrophages. This contributes to the stabilization of atherosclerotic plaques [93]. Corilagin, GZTLT, and FTZ have shown efficacy in preclinical models by inhibiting NLRP3 inflammasome activation and reducing macrophage pyroptosis, thereby slowing AS progression. However, challenges such as optimizing bioavailability, ensuring dosage standardization, and addressing variability in patient responses remain critical for their clinical translation. The exosomal long noncoding RNA AU020206 reduces macrophage pyroptosis by suppressing CEBPB-mediated NLRP3 transcription and lowering caspase-1 activation and IL-1β release, thereby limiting inflammation [94]. Despite its promise, clinical translation faces challenges in optimizing exosome delivery and ensuring consistent therapeutic effects across patient populations. Melatonin reduces macrophage pyroptosis by modulating the SIRT3/FOXO3α/ROS axis, inhibiting NLRP3 inflammasome activation, and lowering IL-1β release [95]. Apigenin inhibits macrophage pyroptosis by suppressing the NLRP3 inflammasome and the MAPK/NF-κB pathway, reducing oxidative stress, caspase-1 activation, and the release of IL-1β and IL-18, thereby contributing to the stabilization of atherosclerotic lesions [96]. Polydatin has therapeutic effects on AS by inhibiting NLRP3 inflammasome-mediated pyroptosis in macrophages, reducing caspase-1 activation and GSDMD cleavage, and promoting autophagy via the NLRP3/mTOR pathway, ultimately diminishing inflammation and enhancing plaque stability [97]. Preclinical studies have demonstrated its efficacy, but challenges for clinical translation include optimizing bioavailability and establishing safe, effective dosing regimens. Further research is needed to ensure consistent therapeutic outcomes and safety across diverse patient populations. TRIM64 exacerbates AS by promoting macrophage pyroptosis through NLRP3 inflammasome activation driven by NF-κB signaling via IκBα ubiquitination, making it a promising therapeutic target for reducing plaque progression and instability [98]. miR-223 inhibits NLRP3-mediated macrophage pyroptosis by downregulating NLRP3, reducing Caspase-1 activation and inflammatory cytokine release. This decreases inflammation and stabilizes atherosclerotic plaques, making miR-223 a promising therapeutic target for the treatment of AS [99]. Targeting the TXNIP/NLRP3 pathway can mitigate nicotine-induced AS by reducing macrophage pyroptosis and associated inflammation [100]. Targeting HDAC6 mitigates nicotine-induced macrophage pyroptosis by inhibiting the NF-κB/NLRP3 signaling pathway. This inhibition reduces the deacetylation and nuclear translocation of p65, ultimately downregulating NLRP3 expression and suppressing the activation of caspase-1. Consequently, the release of the proinflammatory cytokines IL-1β and IL-18 is diminished, thereby alleviating inflammation and promoting plaque stability in AS [101]. MCC950 effectively treats AS by inhibiting NLRP3-mediated macrophage pyroptosis, reducing inflammation, and stabilizing plaques [102]. MCC950 has shown significant potential in preclinical studies because it can reduce atherosclerotic lesions and inflammation, yet its clinical translation remains challenging owing to patient response variability and the need to optimize dosing to ensure efficacy while minimizing off-target effects. Targeting hematopoietic cell kinase may reduce AS by inhibiting NLRP3-driven macrophage pyroptosis, thereby lowering inflammation and stabilizing plaques [103]. Activation of ALDH2 reduces AS by inhibiting ox-LDL-induced NLRP3 inflammasome priming and activation in macrophages, which attenuates oxidative stress, decreases caspase-1 activation and IL-1β release, and mitigates macrophage pyroptosis, ultimately leading to reduced inflammation and enhanced plaque stability [87]. SA reduces AS by downregulating lncRNA-MALAT1, which suppresses NLRP3 inflammasome activation in macrophages, leading to decreased caspase-1 activation and reduced release of proinflammatory cytokines such as IL-1β [79]. Magnesium chloride inhibits the TLR4/NF-κB pathway, reducing NLRP3 activation and IL-1β release, which slows macrophage pyroptosis and AS progression [104]. 

Several therapeutic agents targeting the NLRP3 inflammasome have demonstrated potential in reducing macrophage pyroptosis and slowing AS progression. Compounds such as Mito-Tempo, ATO, Corilagin, GZTLT, FTZ, the exosomal long noncoding RNA AU020206, melatonin, apigenin, and polydatin have shown efficacy in preclinical models by inhibiting NLRP3 activation, reducing caspase-1 activation, and decreasing the levels of proinflammatory cytokines such as IL-1β and IL-18, thus stabilizing atherosclerotic plaques. However, challenges related to bioavailability, off-target effects, and patient variability continue to impede clinical translation. Novel molecular targets, including TRIM64, miR-223, and the TXNIP/NLRP3 pathway, provide additional therapeutic opportunities. While preclinical results are encouraging, further research is needed to refine dosing, confirm long-term safety, and ensure consistent efficacy across patient populations. Addressing these hurdles is crucial for advancing NLRP3-targeted therapies from experimental models to clinical application in AS management.

### Caspase-1-related therapeutic targets

Caspases are essential proteases that govern cell death and inflammation and act as key regulators of macrophage pyroptosis. Their activation drives inflammatory cell death, making them crucial therapeutic targets for the modulation of immune responses and inflammation [122]. Hydrogen sulfide inhibits macrophage pyroptosis by sulfhydrating caspase-1, thereby reducing its activation and cleavage. This suppression decreases IL-1β and IL-18 release, stabilizing atherosclerotic plaques and slowing disease progression [105]. 1G244 targets caspase-1 to induce M1 macrophage pyroptosis, thereby reducing plaque size while preserving M2 macrophages and increasing collagen levels, thus increasing plaque stability [106]. VX765, by inhibiting caspase-1, reduces macrophage pyroptosis and inflammation and stabilizes atherosclerotic plaques. It blocks NLRP3 inflammasome assembly, decreases IL-1β production, and promotes mitophagy and efferocytosis, indicating significant therapeutic potential in AS [107]. lncRNA H19 reduces AS by inhibiting caspase-1-mediated macrophage pyroptosis. This inhibition is achieved by downregulating the expressions of NLRP3, ASC, GSDMD, and cleaved caspase-1, which in turn reduces the release of proinflammatory cytokines such as IL-1β and IL-18. Additionally, the lncRNA H19 suppresses the NF-κB pathway, mitigates mitochondrial dysfunction, and decreases oxidative stress, contributing to reduced inflammation and increased plaque stability [108]. Z-YVAD-FMK mitigates AS by inhibiting caspase-1-mediated pyroptosis, leading to reduced inflammation and macrophage infiltration, as well as plaque stabilization through the suppression of IL-1β and VCAM-1 expression [109]. 

Therapies such as H _2_S, 1G244, VX765, lncRNA H19, and Z-YVAD-FMK have shown significant potential in preclinical models by inhibiting caspase-1, reducing inflammation, and stabilizing plaques. H _2_S and VX765 suppress IL-1β and IL-18 release, whereas 1G244 promotes plaque stability by preserving M2 macrophages. The lncRNAs H19 and Z-YVAD-FMK also inhibit caspase-1-mediated pyroptosis, further reducing inflammation. Despite these advances, challenges such as optimizing dosing and bioavailability and ensuring long-term safety remain, necessitating further research to refine their clinical applicability. 

### GSDMD-related therapeutic targets

As a hallmark of pyroptosis, interventions targeting GSDMD have garnered significant interest because of their potential in treating various diseases, particularly AS [123]. Inhibitors, such as GI-Y1, combat AS by blocking GSDMD-induced pyroptosis in macrophages. These drugs prevent mitochondrial pore formation and DNA release, which inhibits the cGAS-STING-IRF3/NF-κB pathway, reducing IL-1β secretion and limiting inflammation and macrophage accumulation in plaques, ultimately slowing disease progression [110]. Disulfiram (DSF) inhibits macrophage pyroptosis by blocking the formation of GSDMD pores, which prevents the release of IL-1β and reduces inflammation. This slows the progression of atherosclerotic plaques and enhances their stability. In addition, DSF induces autophagy, improves efferocytosis, and alters the gut microbiota, collectively contributing to its anti-atherosclerotic effects [111]. Z-LLSD-FMK treats AS by inhibiting GSDMD-mediated pyroptosis, reducing inflammation and macrophage infiltration, and stabilizing plaques through decreased IL-1β and VCAM-1 levels [109]. 

Necrosulfonamide (NSA)-loaded nanoparticles inhibit GSDMD-mediated pyroptosis in macrophages by blocking the formation of GSDMD pores, which prevents the release of the proinflammatory cytokines IL-1β and IL-18. This action significantly reduces inflammation and stabilizes atherosclerotic plaques. Targeting NSA directly to macrophages via porous nanoparticles enhances the effectiveness of the drug while minimizing its toxicity, offering a promising therapeutic approach to combatting AS [112]. Recent studies have explored various nanoparticle platforms for targeting macrophages in AS, such as melanin nanoparticles functionalized for MR imaging-guided delivery, which have been shown to attenuate pyroptosis through ROS scavenging and the downregulation of pyroptosis-related proteins [124]. Additionally, strategies utilizing siRNA-loaded nanoparticles targeting CaMKIIγ in lesional macrophages have demonstrated improved plaque stability, decreased necrotic core areas, and enhanced efferocytosis in preclinical models [125]. These findings, combined with efforts to use ultrasmall nanoparticles to infiltrate pyroptotic macrophages via GSDMD-mediated membrane pores, emphasize the importance of nanoparticle size and surface properties for efficient cytosolic delivery [126]. Challenges in the application of NSA-loaded nanoparticles include overcoming off-target effects and minimizing toxicity. Despite their high therapeutic potential, nanoparticle-mediated therapies must ensure efficient cellular uptake, escape from endosomal entrapment, and long-term biocompatibility to prevent unintended systemic inflammation or immune activation. Targeting NSA specifically to macrophages via porous nanoparticles not only increases the efficacy of the drug but also offers a promising approach to mitigate these challenges and enhance the safety profile for long-term AS treatment. 

### Other pyroptosis-related therapeutic targets

Macrophage-derived EVs carrying siHMGB1 prevent AS by inhibiting caspase-11-dependent pyroptosis. These EVs downregulate HMGB1, reducing caspase-11 activation and subsequent GSDMD cleavage, which curtails IL-1β and IL-18 release, decreases inflammation, and limits macrophage-driven plaque progression [113]. TRIM25 promotes AS by inducing macrophage pyroptosis through XRCC1 ubiquitination and PARP1 activation, leading to increased M1 polarization and inflammation, which accelerates plaque progression [114]. Targeting LINC01272, which regulates the miR-515/KLF6 axis to activate NLRP3, has therapeutic potential for reducing macrophage pyroptosis and slowing AS progression [115]. Quercetin protects against AS by inhibiting macrophage pyroptosis. It binds to KEAP1 at Arg483 and activates the NRF2 pathway, which reduces oxidative stress and suppresses NLRP3 inflammasome activation, thereby stabilizing plaques and decreasing inflammation [116]. Exogenous spermine inhibits macrophage pyroptosis by activating the NRF2 pathway, thereby reducing oxidative stress and activating the NLRP3 inflammasome. This action decreases inflammation and stabilizes plaques, suggesting a potential treatment for diabetic AS [117]. Electrical stimulation (ES) activates sirtuin 3, reducing macrophage pyroptosis and oxidative stress, which stabilizes atherosclerotic plaques, making ES a promising noninvasive therapy for AS [118]. NIX-mediated mitophagy reduces AS by inhibiting macrophage pyroptosis, thereby reducing inflammation and stabilizing plaques [119]. 

### Exploring alternative pathways and interdisciplinary collaboration

Although targeting macrophage pyroptosis through pathways such as the NLRP3 inflammasome, caspase-1, and GSDMD represents a promising therapeutic approach for AS, other potential treatment directions deserve attention to broaden the scope of AS management. Exploring additional cell death mechanisms, such as ferroptosis and autophagy, may complement pyroptosis inhibition and offer comprehensive strategies for enhancing plaque stability. Recent studies have highlighted the interaction between pyroptosis and ferroptosis in AS, suggesting that therapies targeting multiple pathways can improve treatment efficacy [127]. Interdisciplinary collaboration involving nanotechnology and biomaterials is becoming increasingly critical for the advancement of targeted therapies. Innovations such as melanin nanoparticles and CpG-conjugated silver nanoparticles have demonstrated the potential to enhance drug delivery, reduce inflammation, and stabilize atherosclerotic plaques, underscoring the importance of cross-field integration in the development of future AS treatments [ 124, 128] . In summary, expanding beyond pyroptosis-targeted therapies, incorporating diverse cell death mechanisms and interdisciplinary innovations can significantly enhance the therapeutic outcomes for AS and broaden our understanding of its treatment possibilities. 

**Table:** Table 1 Therapeutic strategies targeting macrophage pyroptosis in AS

Target	Therapeutic agent/strategy	Type	Mechanism	Reference
NLRP3	Mito-Tempo	Mitochondria-targeted antioxidant	Inhibits NLRP3 inflammasome activation by modulating the Nrf2/NLRP3 signaling pathway	[89]
	ATO	Small molecule compound	Inhibits CD36-mediated ox-LDL uptake, suppresses TLR4/NF-κB signaling, and downregulates NLRP3 and caspase-1 activation	[90]
	Corilagin	Polyphenol compound	Inhibits NLRP3 inflammasome activation via the Olfr2 signaling pathway	[91]
	GZTLT	Herbal formula	Inhibits Piezo1-mediated NLRP3 inflammasome activation	[92]
	FTZ	Herbal formula	Inhibits NLRP3 inflammasome activation by reducing oxidative stress through the suppression of ROS production, protecting mitochondrial integrity	[93]
	lncRNA-AU020206	RNA therapy	Suppresses CEBPB-mediated NLRP3 transcription	[94]
	MT	Neuroendocrine hormone therapy	Regulates the SIRT3/FOXO3α/ROS axis	[95]
	API	Flavonoid compound	Inhibits NLRP3 inflammasome activation by suppressing ROS and the MAPK/NF-κB pathway	[96]
	Polydatin	Natural polyphenol compound	Inhibits NLRP3 inflammasome activation by reducing ROS and downregulating mTOR, promoting autophagy to prevent inflammasome assembly	[97]
	TRIM64 inhibitor	Gene silencing	Inhibits NF-κB pathway via IκBα ubiquitination	[98]
	miR-223	MicroRNA therapy	Binds to the 3′UTR of NLRP3 mRNA, blocking its translation	[99]
	TXNIP inhibitor	Gene silencing	Inhibits ROS-TXNIP/NLRP3 pathway	[100]
	HDAC6 inhibitor	Histone deacetylase inhibitor	Suppresses the HDAC6/NF-κB/NLRP3 pathway by reducing p65 deacetylation, preventing its nuclear translocation and subsequent NLRP3 transcription.	[101]
	MCC950	Selective NLRP3 inflammasome inhibitor	Inhibits NLRP3/ASC/Caspase-1/GSDMD axis	[102]
	HCK inhibitor	Non-receptor tyrosine kinase inhibitor	Inhibits NLRP3 inflammasome activation by blocking HCK, which reduces ASC oligomerization and caspase-1 activation	[103]
	Alda-1	Enzyme activator	Inhibits oxidized LDL-induced NLRP3 inflammasome priming and activation by reducing oxidative stress through ROS attenuation and decreasing 4-HNE levels	[87]
	SA	Natural phenolic acid	Downregulates lncRNA-MALAT1 and inhibits NLRP3 inflammasome	[79]
	MgCl _2_	Inorganic compound	Inhibits TLR4/NF-κB signaling, reduces NLRP3-mediated pyroptosis	[104]
Caspase-1	H2S	Gasotransmitter therapy	Inhibits ox-LDL-induced pyroptosis by S-sulfhydrating caspase-1, reducing its activity and blocking the NLRP3/caspase-1/GSDMD pathway	[105]
	1G244	Small molecule inhibitor	Induces NLRP1b-mediated caspase-1 activation, leading to macrophage pyroptosis, which reduces pro-inflammatory M1 macrophages and stabilizes atherosclerotic plaques	[106]
	VX765	Caspase-1 inhibitor	Inhibits NLRP3 inflammasome activation by blocking caspase-1, which reduces ASC oligomerization and prevents mitochondrial damage, while promoting mitophagy and efferocytosis	[107]
	LncRNA H19	lncRNA therapy	Mitigates ox-LDL-induced pyroptosis by downregulating caspase-1, inhibiting NLRP3, and reducing NF-κB activation	[108]
	Z-YVAD-FMK	Caspase-1 inhibitor	Inhibits caspase-1, preventing GSDMD activation	[109]
GSDMD	GI-Y1	Small molecule inhibitor	Inhibits GSDMD activation, preventing mitochondrial perforation and suppressing the STING-IRF3/NF-κB signaling axis	[110]
	DSF	Small molecule inhibitor	Inhibits GSDMD-mediated pyroptosis by blocking pore formation, while enhancing autophagy, efferocytosis, and promoting atheroprotective gut microbiota	[111]
	Z-LLSD-FMK	GSDMD inhibitor	Inhibits GSDMD activation by blocking its cleavage	[109]
	NSA	GSDMD inhibitor	Inhibits GSDMD-mediated IL-1β release by binding to GSDMD, preventing pore formation in macrophages	[112]
Other	siHMGB1	RNA interference	Inhibits Caspase-11-dependent pyroptosis by downregulating HMGB1	[113]
	TRIM25 inhibitor	Ubiquitin E3 ligase inhibitor	Reduces macrophage M1 polarization and pyroptosis by inhibiting XRCC1 ubiquitination	[114]
	Targeting LINC01272	Non-coding RNA regulation	Reduces macrophage pyroptosis via LINC01272/miR-515/KLF6 axis	[115]
	Quercetin	Natural antioxidant	Binds KEAP1 to activate NRF2, reducing macrophage pyroptosis and oxidative stress	[116]
	Spermine	Natural polyamine	Activates Nrf2 pathway, reducing oxidative stress and pyroptosis	[117]
	ES	Non-invasive therapy	Activates Sirtuin3, promoting autophagy and reducing ROS, which inhibits pyroptosis	[118]
	NIX activation	Mitochondrial autophagy modulator	Promotes mitophagy, reducing ROS and pyroptosis	[119]

## Conclusions and Prospects

Macrophage pyroptosis in AS pathogenesis is increasingly recognized as a pivotal factor in plaque formation and destabilization. As a form of inflammatory cell death, pyroptosis exacerbates local inflammation within atherosclerotic plaques, driving plaque instability and increasing the risk of rupture, which can lead to severe cardiovascular events. The molecular mechanisms underlying this process, particularly the activation of the NLRP3 inflammasome and subsequent caspase-1-mediated cleavage of GSDMD, highlight potential therapeutic targets for mitigating AS progression.

Recent studies have highlighted the therapeutic potential of targeting macrophage pyroptosis in AS. The inhibition of key components of the pyroptosis pathway, such as NLRP3, caspase-1, and GSDMD, has shown promise in preclinical models, suggesting that these interventions can effectively reduce inflammation, stabilize plaques, and prevent adverse cardiovascular outcomes. For example, the genetic elimination of GSDMD or pharmacological inhibition of its activity has been demonstrated to delay AS progression by reducing inflammatory cell death and neutrophil extracellular trap formation, which are critical in the pathophysiology of the disease [ 8, 129] . 

Compared with other cell death pathways, such as apoptosis and necroptosis, pyroptosis has unique advantages. Pyroptosis directly drives inflammation through the release of proinflammatory cytokines such as IL-1β and IL-18; thus inhibiting pyroptosis not only prevents cell death but also reduces the inflammatory cascade central to AS progression. In contrast, apoptosis, which effectively reduces the number of cells within plaques, may lead to the formation of secondary necrotic cells if efferocytosis is impaired, which can exacerbate inflammation. Necroptosis, another regulated form of necrosis, shares some inflammatory features with pyroptosis but may not offer the same specific inflammatory control as targeting the NLRP3-caspase-1-GSDMD axis, a major driver of AS-related inflammation. Nevertheless, pyroptosis-targeted therapies have limitations. A potential challenge is the broad inhibition of inflammatory responses, which can impair immune surveillance and the ability to respond to infections. Additionally, patient variability in the response to inhibitors targeting these pathways could pose challenges for personalized treatment approaches. In contrast, apoptosis-targeted therapies may offer a safer profile in terms of immune modulation; however, they may be less effective in halting the inflammatory amplification caused by pyroptosis. Therefore, a comprehensive approach combining pyroptosis inhibition with strategies aimed at modulating apoptosis and necroptosis may yield improved therapeutic outcomes.

Novel therapeutic approaches, including the use of nanoparticle-mediated delivery systems, are being explored to increase the specificity and efficacy of antipyroptotic agents. These nanotechnologies offer a promising avenue for overcoming the challenges associated with drug delivery, such as poor bioavailability and off-target effects, by enabling the precise targeting of pyroptotic pathways within atherosclerotic lesions. Such advancements could revolutionize the treatment landscape of AS, providing new hope for patients at high risk of cardiovascular events [130]. 

Future research should focus on refining these therapeutic strategies with an emphasis on clinical translation. Understanding the complex interplay between the different forms of programmed cell death, including pyroptosis, apoptosis, and necroptosis, is essential for developing comprehensive treatment regimens that address the multifactorial nature of AS. Additionally, the identification of novel pyroptosis-related biomarkers could facilitate early diagnosis and personalized treatment, further improving patient outcomes.

In conclusion, targeting macrophage pyroptosis is a promising therapeutic strategy for AS management. However, combining pyroptosis inhibition with therapies that address other cell death pathways, alongside the development of advanced drug delivery technologies, may significantly reduce the burden of cardiovascular disease by providing a more holistic approach to AS treatment.
